# Prophage Tracer: precisely tracing prophages in prokaryotic genomes using overlapping split-read alignment

**DOI:** 10.1093/nar/gkab824

**Published:** 2021-09-22

**Authors:** Kaihao Tang, Weiquan Wang, Yamin Sun, Yiqing Zhou, Pengxia Wang, Yunxue Guo, Xiaoxue Wang

**Affiliations:** Key Laboratory of Tropical Marine Bio-resources and Ecology, Guangdong Key Laboratory of Marine Materia Medica, Innovation Academy of South China Sea Ecology and Environmental Engineering, South China Sea Institute of Oceanology, Chinese Academy of Sciences, No. 1119, Haibin Road, Nansha District, Guangzhou 511458, China; Southern Marine Science and Engineering Guangdong Laboratory (Guangzhou), No. 1119, Haibin Road, Nansha District, Guangzhou 511458, China; Key Laboratory of Tropical Marine Bio-resources and Ecology, Guangdong Key Laboratory of Marine Materia Medica, Innovation Academy of South China Sea Ecology and Environmental Engineering, South China Sea Institute of Oceanology, Chinese Academy of Sciences, No. 1119, Haibin Road, Nansha District, Guangzhou 511458, China; Southern Marine Science and Engineering Guangdong Laboratory (Guangzhou), No. 1119, Haibin Road, Nansha District, Guangzhou 511458, China; University of Chinese Academy of Sciences, Beijing, China; Research Center for Functional Genomics and Biochip, 23 Hongda St., Tianjin 300457, China; Key Laboratory of Tropical Marine Bio-resources and Ecology, Guangdong Key Laboratory of Marine Materia Medica, Innovation Academy of South China Sea Ecology and Environmental Engineering, South China Sea Institute of Oceanology, Chinese Academy of Sciences, No. 1119, Haibin Road, Nansha District, Guangzhou 511458, China; Southern Marine Science and Engineering Guangdong Laboratory (Guangzhou), No. 1119, Haibin Road, Nansha District, Guangzhou 511458, China; University of Chinese Academy of Sciences, Beijing, China; Key Laboratory of Tropical Marine Bio-resources and Ecology, Guangdong Key Laboratory of Marine Materia Medica, Innovation Academy of South China Sea Ecology and Environmental Engineering, South China Sea Institute of Oceanology, Chinese Academy of Sciences, No. 1119, Haibin Road, Nansha District, Guangzhou 511458, China; Southern Marine Science and Engineering Guangdong Laboratory (Guangzhou), No. 1119, Haibin Road, Nansha District, Guangzhou 511458, China; University of Chinese Academy of Sciences, Beijing, China; Key Laboratory of Tropical Marine Bio-resources and Ecology, Guangdong Key Laboratory of Marine Materia Medica, Innovation Academy of South China Sea Ecology and Environmental Engineering, South China Sea Institute of Oceanology, Chinese Academy of Sciences, No. 1119, Haibin Road, Nansha District, Guangzhou 511458, China; Southern Marine Science and Engineering Guangdong Laboratory (Guangzhou), No. 1119, Haibin Road, Nansha District, Guangzhou 511458, China; University of Chinese Academy of Sciences, Beijing, China; Key Laboratory of Tropical Marine Bio-resources and Ecology, Guangdong Key Laboratory of Marine Materia Medica, Innovation Academy of South China Sea Ecology and Environmental Engineering, South China Sea Institute of Oceanology, Chinese Academy of Sciences, No. 1119, Haibin Road, Nansha District, Guangzhou 511458, China; Southern Marine Science and Engineering Guangdong Laboratory (Guangzhou), No. 1119, Haibin Road, Nansha District, Guangzhou 511458, China; University of Chinese Academy of Sciences, Beijing, China

## Abstract

The life cycle of temperate phages includes a lysogenic cycle stage when the phage integrates into the host genome and becomes a prophage. However, the identification of prophages that are highly divergent from known phages remains challenging. In this study, by taking advantage of the lysis-lysogeny switch of temperate phages, we designed Prophage Tracer, a tool for recognizing active prophages in prokaryotic genomes using short-read sequencing data, independent of phage gene similarity searching. Prophage Tracer uses the criterion of overlapping split-read alignment to recognize discriminative reads that contain bacterial (*attB*) and phage (*attP*) *att* sites representing prophage excision signals. Performance testing showed that Prophage Tracer could predict known prophages with precise boundaries, as well as novel prophages. Two novel prophages, dsDNA and ssDNA, encoding highly divergent major capsid proteins, were identified in coral-associated bacteria. Prophage Tracer is a reliable data mining tool for the identification of novel temperate phages and mobile genetic elements. The code for the Prophage Tracer is publicly available at https://github.com/WangLab-SCSIO/Prophage_Tracer.

## INTRODUCTION

Temperate phages can integrate into the bacterial chromosome to become prophages and enter lysogeny, maintaining a long-term association with their bacterial hosts. Lysogeny may be more prevalent than lytic cycles in bacteria-phage interactions and may become increasingly important in ecosystems with high microbial densities (1,2). Majority of commensal bacteria within the human and murine gut, as well as in coral microbiota (3–5), were found to be lysogens, and prophages can be spontaneously induced as active phages (4). Prophages may constitute up to 20% of a bacterium's genome (6) and serve as regulatory switches that regulate bacterial genes via genome excision (7,8). A novel family of non-tailed dsDNA viruses, *Autolykiviridae*, was identified recently and revealed a large number of previously unrecognized prophages in various bacterial taxa (9). Although the metagenomic analysis of geographically diverse samples contributes to the identification of new viruses (10,11), identifying novel prophages in prokaryotic genomes remains challenging.

Many tools have been developed to predict prophages using various strategies (12–18). Most of these methods, including Phage_Finder, PHASTER, VirSorter and Prophage Hunter, are mainly dependent on sequence similarity searching against a built-in validated dataset containing known phages to recognize phage-related gene enriched regions. However, phages are highly divergent and evolve rapidly. Sequence conservation among phage structural proteins, such as major capsid proteins (MCPs), decreases rapidly, even over short evolutionary distances (19,20), and therefore may not indicate readily detectable similarity with identified phages. In addition, known phages may represent only a small portion of phage diversity (10,11), and a previous analysis demonstrated that most identified prophages are derived from a small number of host phyla (21). Furthermore, auxiliary metabolic genes are prevalent in phages (11,22,23), which may also blur the boundaries between prophages and host genome sequences. Therefore, sequence-similarity-independent approaches are needed to identify novel temperate phages.

Compared to obligate lytic phages, the life cycle of temperate phages includes a lysis-lysogeny decision-making process. The lytic conversion of active prophages can affect individual cells, as well as entire communities, and is central to bacterial physiology, metabolism and evolution. Cryptic prophages, which are incapable of forming plaques, can also provide multiple benefits to the host for surviving adverse environmental conditions (24). We previously discovered that the cryptic prophage CP4So in *Shewanella oneidensis* excises specifically to increase the survival of host at cold temperatures (25), and recently we further revealed that the excision of CP4So relies on temperature-dependent phosphorylation of the host H-NS (26). Indeed, the spontaneous induction of various prophages at low rates has been observed in various bacterial taxa (27–29). Moreover, stress conditions, such as UV and oxidative stress, and biofilm formation also trigger prophage induction and/or prophage excision (24,30,31). Conventional whole-genome sequencing or the resequencing of microbes can generate millions of pieces of short-read or long-read DNA sequencing data. Among these reads, a large number are not properly aligned when mapped to the reference genomes, which may be attributable to horizontal gene transfer, genome rearrangement, and the activities of mobile DNA elements (32). These improperly aligned reads, including split reads and discordant read pairs, are usually overlooked during the genome assembly process. However, they may provide extra information on prophage induction and/or excision. Therefore, we reasoned that the split reads generated from prophage induction and/or prophage excision may provide an important genetic resource to identify unknown prophages hidden in various microbial hosts.

Therefore, we designed Prophage Tracer, a simple algorithm that uses overlapping split-read alignment to identify active and cryptic prophages hidden in DNA sequencing data. The basic logic of Prophage Tracer is that the attachment sites of direct repeats (*attL* and *attR*) are recombined to form bacterial (*attB*) and phage (*attP*) *att* sites (*att* sites representing *attL*/*R/B*/*P* common core sequences), and reads containing *attB* or *attP* can generate overlapping split-read alignments. These discriminative signals can facilitate the prediction of prophages, requiring a minimum of only one split read. In this study, utilizing the simulated reads and DNA sequencing reads of a variety of bacterial species, we demonstrate that Prophage Tracer can predict known and novel active prophages that are highly diverse with precise boundaries. This approach is independent of phage gene similarity search. Taking advantage of DNA sequencing data, Prophage Tracer is a reliable data mining tool and is complementary to other current state-of-the-art tools for the study of prophages.

## MATERIALS AND METHODS

### Prophage workflow

For the chromosome-level assembled genome, split reads and discordant read pairs were extracted from the alignment in SAM (Sequence Alignment/Map) format generated by Burrows-Wheeler Aligner (BWA-mem algorithm) (33,34). Split reads cannot be represented as a linear alignment that can be split into more than two parts that are aligned to different parts of the reference genome. First, split reads were preliminarily extracted according to FLAG strings matching *a*S*b*M and CIGAR strings matching 145, 81, 99 or 163 or FLAG strings matching *a*M*b*S and CIGAR strings matching 97, 161, 147 or 83. The integer values of *a* and *b* were allowed from 10–150 for paired-end reads (2 × 150 bp) generated by commonly used Illumina instruments. These reads were extracted for further BlastN (35) searching against the reference genome. If one read split into two parts spanning *R_1_*–*R_2_* and *R_3_*–*R_4_* on the query read, the integer values of these locations should be *R_1_* <*R_3_* <*R_2_* <*R_4_* and were aligned to two different regions of the reference genome by BlastN, ensuring an overlapping split-read alignment. Reads containing *attB* or *attP* can be differentiated by the FLAG strings and the alignment locations on the reference genomes. The *R_1_* to *R_4_* locations represent the endpoints of *attL* and *attR* of prophage candidates. These filtered reads were subsequently clustered and summarized according to the *R_1_* to *R_4_* locations. Furthermore, discordant read pairs were extracted according to FLAG strings matching *d*M (integer values of *d* > 130) and CIGAR strings matching 97, 145, 81 or 161 and merged to the previously clustered split reads according the values of POS and MRNM fields in the SAM file and whether they spanned the *R_1_* to *R_4_* locations. The positions between discordant read pairs representing *attB* and *attP* were also considered in the clustering process. The positions of representative extracted discordant read pairs are shown in Supplementary Figure S1. Finally, prophage candidates were filtered according to *att* site length (default > 2 bp), prophage size (default >5000 and <150 000 bp), and *attB*/*attP* event count (default both ≥1). The default parameters of *att* site length and prophage size were established according to previous studies (17,18).

For contig-level assembled genomes, further steps were employed to extract split reads and discordant read pairs. Briefly, if an intact prophage was located in two separate contigs, in consideration of four possible orientations, FLAG strings matching *a*S*b*M and CIGAR strings matching 113 or 117 or FLAG strings matching *a*M*b*S and CIGAR strings matching 65 or 129 were further used to extract split reads. FLAG strings matching *d*M and CIGAR strings matching 177, 113, 129 or 65 were further used to extract discordant read pairs.

### Comparison with LUMPY using simulated data

To simulate genomes containing prophages, we used a custom shell script available via Prophage Tracer GitHub (https://github.com/WangLab-SCSIO/Prophage_Tracer). Genomes with ∼4 M base pairs containing one prophage each were simulated. The length of the *att* site was randomly selected from 2 to 145 bp (with a 1–2 bp mismatch if *att* site > 2 bp) and prophage size from 5000 to 150 000 bp. The GC content across genomes was allowed to be 20–80%. The corresponding bacterial host genomes with prophage-excised (containing *attB*) and circular prophage genomes (containing *attP*) were also generated. Paired reads of 2 × 150-bp with four different sequencing depths (10×, 20×, 50× and 100×) were generated using the sequencing read simulator GemSIM (36) in metagenomic mode which was used to simulate four different ratios of the host genome, host genome with prophage excised, and circular prophage genome (WT: *attB*: *attP*). A total of 320 sequencing read data points from 20 genomes were simulated, and this step was repeated three times. Simulated sequencing reads were aligned to reference genomes by Burrows-Wheeler Aligner (BWA-MEM algorithm) (33,34), and duplicates were removed by sambamba (37). The outputs were further compared to evaluate the effect of sequencing depth on the sensitivity of LUMPY and Prophage Tracer at various sequencing depths or *att* site lengths. The default parameters and pre-processing steps of data used in LUMPY procedure were the same as indicated on the LUMPY GitHub (https://github.com/hall-lab/lumpy-sv).

### Identification and characterization of prophages in coral-associated bacteria

The genomes of seven bacteria belonging to Alphaproteobacteria, Gammaproteobacteria, and Flavobacteriia were sequenced by the Illumina and PacBio platforms, and complete genomes were assembled and annotated by the NCBI Prokaryotic Genome Annotation Pipeline (38). Short-read data from Illumina were used to predict active prophages with Prophage Tracer, and genome sequences were analyzed using the LUMPY, PHASTER and Prophage Hunter web portals. The prophage excision and predicted *attB* and *attP* sites were confirmed by a PCR-based assay followed by sequencing using primers flanking each prophage (Supplementary Table S1). The prophage excision rate was evaluated by quantitative PCR (qPCR) as previously described (25). The relative amounts of the excised prophages were determined using the reference gene *gyrB*. qPCR was assayed for technical triplicates of each biological repeat. Primer pairs are listed in Supplementary Table S1. Sequencing depths (i.e. coverage) across the genomes of seven coral-associated bacteria were plotted using karyoploteR with a window size of 1000 bp (39).

### Prediction of prophages in publicly available genomes

Prophage Tracer was tested using publicly available chromosome-level genomes that had their corresponding short-read sequencing data also deposited in NCBI (Supplementary Table S2). In order to evaluate the capability of Prophage Tracer on the chromosome-level and the contig-level of assembled genomes, these genomes were reassembled to the contig-level only using their short-read sequencing data by Shovill v1.1.0 with default parameters (T. Seeman, https://github.com/tseemann/shovill). Short-read sequencing data were pre-processed by Trimmomatic v0.39 (40) to remove low-quality (pred33) regions and adapters. Predicted prophages from two different levels of genome assemblies were manually checked for the presence of phage structural genes or other phage related genes annotated using the CDD database (41). Chromosome-level, contig-level and prophage genomes of each strain were aligned by QUAST v5.0.2 (42) to confirm the locations of contigs and prophages on the chromosomes.

### Phylogenetic analysis

Each sequence of major capsid protein of representative prophages was used as a query for PSI-BLAST (43) against the NR database and sequences with e-value < 0.05 were collected. All recovered sequences were clustered at 70% identity using CD-HIT suite (44).The filtered sequences were aligned by MAFFT (45) and further edited by trimAl (46). Each final data set was used for the maximum likelihood (ML) phylogenetic analysis by the W-IQ-TREE (47). The best-fit substitution model was automatically determined and the reliability of internal branches was tested by 1000 ultrafast bootstrap replicates (48) in the W-IQ-TREE web interface. The tree was further annotated by the iTOL tool (49).

## RESULTS

### Overview of Prophage Tracer

Prophage Tracer employs a simple principle: prophage induction and/or excision can generate genetic structural variations, including circular prophage DNAs and/or large genomic deletions on the bacterial chromosome. This process leads to some sequencing reads being improperly aligned to the reference genome during genome assembly. These improperly aligned reads can be utilized to identify prophages and to locate prophage boundaries. This strategy does not rely on known phage sequences and has the potential to identify novel prophages.

**Figure 1.**
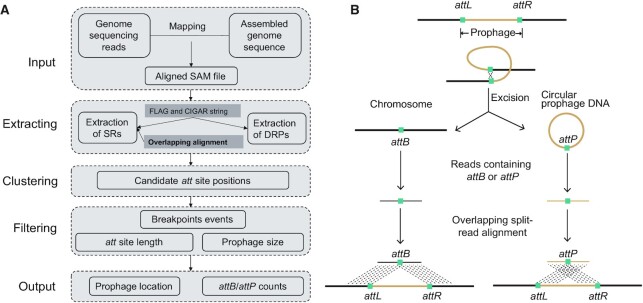
Prophage Tracer workflow using overlapping split-read alignment to detect prophages. (**A**) The workflow schematics of Prophage Tracer including extracting, clustering and filtering steps. (**B**) Reads containing *attB* or *attP* caused by prophage excision can generate overlapping alignments (overlapping length is approximately equal to *att* sites), which can be a discriminative signal for prophage detection. SRs: split reads; DRPs: discordant read pairs.

The overall Prophage Tracer workflow is shown in Figure 1A. Prophage Tracer takes aligned reads in SAM format as input. First, split reads and discordant read pairs are preliminarily extracted according to FLAG and CIGAR strings (defined by the SAM specification). As illustrated in Figure 1B, if the split reads contain *attB* or *attP* sites, then this site matches the *attL* and *attR* of the reference genome. Therefore, alignment of the split read and the corresponding reference genomes generate overlapping regions inside the split reads, suggesting that this region contains potential *attB* or *attP* sites. The concept of overlapping split-read alignment is simple but critical for Prophage Tracer to precisely identify candidate prophages. Next, overlapping alignment from BlastN output is used to infer the precise positions of *attL* and *attR* sites (Figure 1B and Supplementary Figure S2A). Candidate prophage boundaries are clustered by judging the proximity of all four of *attL* and *attR* site positions, and discordant read pairs are merged according to the candidate prophage boundaries. Meanwhile, *attB*/*attP* events are counted for each candidate prophage. Finally, candidate prophages are filtered by *attB*/*attP* event count, prophage size and *att* site length.

This approach can eliminate the overwhelming numbers of false positive split reads that are generated in mapping routine bacterial genome sequencing reads by other types of unknown structural variations. This approach can be applied to chromosome- or contig-level genomes. For an intact prophage located in a complete-level genome or in one contig of a contig-level genome, Prophage Tracer can provide the precise positions of *att* sites and the lengths of prophages. For an intact prophage located separately at the termini of two contigs of contig-level genomes, Prophage Tracer can also provide precise positions of *att* sites and the approximate lengths of prophages, which might be useful as a screening tool to determine whether contigs are worth converting contigs to complete-genomes for the extraction of intact prophages. Furthermore, mobile genetic elements that rely on site-specific recombinases can also detected by Prophage Tracer. The requirement of CPU and memory usage for Prophage Tracer is low, and the runtime is ∼30–60 s per run. A typical output of Prophage Tracer contains positions of *attL* and *attR*, evidence counts of *attB* and *attP*, and overlapping split-read alignment to enable the further manual determination of the potential impact on genes disrupted at the integration sites.

### Comparison with LUMPY using simulated data

**Figure 2.**
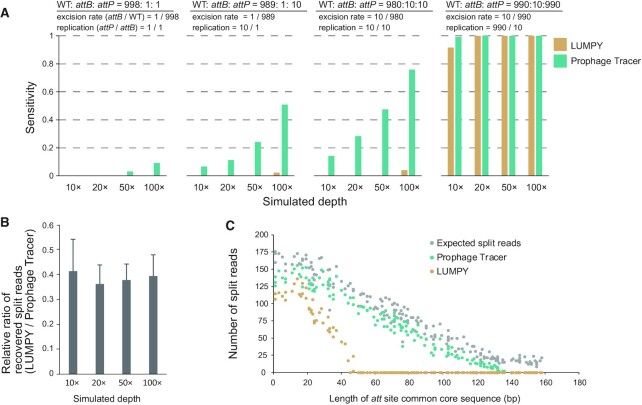
Performance comparison of Prophage Tracer and LUMPY using simulated data. (**A**) Comparison of sensitivity for prophage detection. Sensitivity is defined as the average ratio of positive hits of three rounds of simulated data (each round with 20 genomes). The ratio of the host genome, host genome with prophage excised and circular prophage genome (WT: *attB*: *attP*) is on the top of each panel. (**B**) The average relative ratio of recovered split reads between LUMPY and Prophage Tracer. (**C**) The recovered split reads by Prophage Tracer and LUMPY from simulated data with *att* sites ranging from 2 to160 bp. Expected split reads in the SAM file using simulated data was extracted according to CIGAR strings of *a*MbS or *a*SbM (integer values of *a* and *b* from 1–149) mapping at expected prophage positions. Detailed information on the simulated data is listed in Supplementary Table S3.

Since Prophage Tracer employs a strategy based on the detection of split reads and discordant read pairs, we compare it with LUMPY, which employs a similar strategy (50). LUMPY is designed for the detection of structural variation and is primarily employed for human genome analysis, as well as for bacterial resequencing analysis (51,52). Overall, Prophage Tracer performed better than LUMPY on simulated data with low prophage excision rates and low sequencing depths (Figure 2). Prophage Tracer was able to detect prophage excision signals when the prophage excision rate (*attB*/WT) was ∼1/1000 (without replication which was calculated from *attP*/*attB*) at a minimum sequencing depth of 50× (left panel of Figure 2A). At this excision rate, if the abundance of circular prophage DNA was 10 times higher, Prophage Tracer could detect prophage excision signals when the sequencing depth was as low as 10× (middle panels of Figure 2A). In comparison, LUMPY required a higher prophage excision rate and a higher abundance of circular prophages, and it only performed as well as with Prophage Tracer when the prophage excision rate (*attB*/WT ≥ 1%) and replication (*attP*/*attB* = 99) were both high (right panel of Figure 2A).

By manually checking the simulated data, we found that the Prophage Tracer could detect more split reads than LUMPY at four different sequencing depths (Figure 2B). Further simulation analysis (using *att* site length 2–160 bp) revealed that Prophage Tracer could extract prophage split reads with *att* site lengths ranging from 2 bp to 130 bp, while LUMPY can only extract the split reads from 2 to 50 bp (Figure 2C and Supplementary Table S3). In addition, the ability to detect split reads by LUMPY was greatly reduced with the increase of *att* site length when *att* site length >20 bp. We further checked the scripts of LUMPY found that the algorithm used by LUMPY to recognize split reads relies on the previously assigned of the ‘SA’ or ‘XA’ tags by BWA-MEM in SAM files. According to the BWA-MEM and the SAM format specification (33,53), an alignment of a read can be linear or chimeric. For a chimeric alignment, it contains a set of alignments that do not have large overlaps. If a chimeric alignment contains two linear alignments spanning *R_1_*–*R_2_* and *R_3_*–*R_4_* on the query read (Supplementary Figure S3), the assignment of ‘SA’ or ‘XA’ tags to the alignment depends on the length the overlaps (*R_2_*–*R_3_*) and the proportion of the overlaps in each linear alignment [(*R_2_* – *R_3_*)/(*R_2_* – *R_1_*) and (*R_2_* – *R_3_*)/(*R_4_* – *R_3_*)]. This limits the ability of LUMPY to detect split reads containing *att* sites larger than 50 bp. Instead, we employed BlastN to generate reliable overlapping alignments from the output of BWA-MEM and a custom algorithm to extract split reads in Prophage Tracer. This strategy enabled Prophage Tracer to precisely detect prophage induction or excision signals as long as the discriminative split reads contain *attB* or *attP*, even with a low prophage excision rate and a low sequencing depth.

### Validation of the Prophage Tracer workflow

To validate the capability of Prophage Tracer to predict prophages, publicly available whole-genome sequencing data of bacterial isolates with identified prophages were utilized. Active and cryptic prophages, including the Pf4 prophage in *Pseudomonas aeruginosa* PAO1, the CP4So and LambdaSo prophages in *S. oneidensis* MR-1, the rac prophage in *Escherichia coli* K-12 and Φ10403S in *Listeria monocytogenes* 10403S, were successfully detected by Prophage Tracer (Table 1 and Supplementary Table S4). Split reads representing the *attP* events of Pf4 were detected, which was consistent with the presence of replicative form Pf4 molecules in the liquid culture of *P. aeruginosa* PAO1 (54). Using published *E. coli* K-12 resequencing data (55), the prophage rac was identified, and only a small number of split reads representing *attP* events were observed in various samples, suggesting that rac can be spontaneously induced at low ratios, which was consistent with the results of our previous research (24,56). Using our resequencing data of *S. oneidensis* MR-1 cultured at 4°C, both the CP4So and LambdaSo prophages were predicted. In contrast, only LambdaSo prophages were predicted at 30°C. This result was in agreement with the results of our previous study, which demonstrated that CP4So was induced only at low temperatures (25) and that LambdaSo had a relatively high excision rate (57). Furthermore, the impact of prophage excision on genes at the integration loci was determined in the Prophage Tracer output. It was demonstrated that the excision of CP4So caused the deletion of a U at the 3′-end of the tmRNA (SsrA), destroying this G·U wobble base pairing (25) (Supplementary Figure S2B). Furthermore, the integration of prophage Φ10403S within *comK* in *L. monocytogenes* 10403S (8,58) was also predicted, and it contains a 3-bp *att* site and a serine-type recombinase. Overall, Prophage Tracer is able to precisely predict known active prophages.

**Table 1.** Prediction of known prophages in four representative strains

Strains	Prophage	Contig	*attL*_start	*attL*_end	*attR*_start	*attR*_end	Size (bp)	Length of *att* site	References
*Pseudomonas aeruginosa* PAO1	Pf4	NC_002516.2	785288	785336	797699	797747	12411	49	(54)
*Shewanella* *oneidensis* MR-1	CP4So	NC_004347.2	1501853	1501946	1538064	1538157	36211	94	(25)
	LambdaSo	NC_004347.2	3074594	3074605	3126435	3126446	51841	12	(57)
*Escherichia coli* K-12	rac	NZ_CP009273.1	1406156	1406198	1429216	1429258	23060	43	(24,55,56)
*Listeria monocytogenes* 10403S	Φ10403S	NC_017544.1	2319845	2319847	2357456	2357458	37611	3	(8,58)

### Comparison with PHASTER/Prophage Hunter/LUMPY to predict prophages

PHASTER (12) and Prophage Hunter (18) are designed for the detection of prophages in prokaryotic genomes using similarity searching. To evaluate the potential of Prophage Tracer to predict prophages, seven different bacterial strains isolated from the stony coral *Galaxea fascicularis* (11,59,60) were sequenced and analyzed by Prophage Tracer and these two methods. In total, nine candidate prophages were predicted by Prophage Tracer (Table 2). In comparison, LUMPY missed four of them because the number of split reads was too low or the length of *att* sites was too long to be detected by LUMPY, which was consistent with our tests on the simulated data above (Supplementary Table S5). In addition, among these nine candidate prophages, PHASTER also identified five of them and Prophage Hunter identified eight of them to some degree (Supplementary Table S5). The annotation of these five prophages demonstrated intact phage structural and regular proteins, such as capsid, head, tail, terminase, portal and integrase (Supplementary Table S6). Next, we checked the boundaries and attachment sites of these prophages using PCR primers to specifically amplify the region containing the *attB* or *attP* region, and we subsequently sequenced these regions (Supplementary Figure S4). The boundaries and attachment sites of these prophages predicted by Prophage Tracer agreed well with the results of the PCR-based assay (Supplementary Table S7). In contrast, some prophage boundaries predicted by Prophage Hunter or PHASTER were not accurate (Table 2).

**Table 2.** Comparison of outputs of the predicted active prophages by Prophage Tracer with PHASTER or Prophage Hunter in seven coral-associated bacterial strains^a^

		Prophage Tracer						
Strain name	Prophage	*attL*_start	*attL*_end	*attR*_start	*attR*_end	Size	PHASTER^b^	Prophage Hunter^c^
*Erythrobacter aquimaris* SCSIO 43205	Pea1	1888722	1888741	1936851	1936870	48129	Questionable (70):	Active (0.9): 1885416–1903092
							1895962–1915899	Active (0.97): 1888722–1936870
								Active (0.91): 1917844–1948048
*Ruegeria conchae* SCSIO 43209	Prc1	1373446	1373460	1379997	1380011	6551	-	Inactive (0.12):1362384–1392851
*Halomonas meridiana* SCSIO 43005	Phm1	292609	292683	333351	333425	40742	Intact (150):	Inactive (0.14): 274307–310741
							293153–331437	Active (0.9): 292613–333425
	Phm2	1064123	1064145	1100156	1100178	36033	Intact (150):	Ambiguous (0.73): 1064268–1100128
							1075299–1101737	
	Phm3	2090511	2090576	2139945	2140010	49434	Incomplete (20):	Active (0.93): 2077440–2104589
							2090437–2116834	Active (0.97): 2090511–2140010
								Ambiguous (0.76): 2124591–2138678
*Vibrio nigripulchritudo* SCSIO 43132 (contig1)	Pvn1	353280	353303	367745	367768	14465	-	Ambiguous (0.77): 350448–374105
*Marixanthomonas ophiurae* SCSIO 43207	Pmo1	2643352	2643371	2676198	2676217	32846	-	-
*Mesoflavibacter sabulilitoris* SCSIO 43206	Pms1	2668021	2668042	2679241	2679262	11220	-	Inactive (0.34): 2648530–2674443
*Zunongwangia mangrovi* SCSIO 43204	Pzm1	1472262	1472314	1512357	1512409	40095	Incomplete (30):	Ambiguous (0.72): 1461300–1484415
							1486303–1511274	Active (0.92): 1469187–1485662
								Active (0.95): 1487346–1517819
								Inactive (0.26): 1510309–1532089

^a^Full outputs of these three tools and LUMPY are shown in Supplementary Table S6.

^b^Outputs of prophage regions predicted by PHASTER (the scores are in parenthesis and the predicted ends are shown). ‘–’ indicates ‘not detected’.

^c^Outputs of prophage regions predicted by Prophage Hunter (the scores are in parenthesis and the predicted ends are shown). ‘–’ indicates ‘not detected’.

Among the five prophages, Phm3 is integrated into the tRNA-Leu of *Halomonas meridiana* SCSIO43005 (Supplementary Table S7). Further analysis showed that this prophage was similar to a metagenomic assembled prokaryotic dsDNA virus (MK892487.1) (Supplementary Figure S5 and Supplementary Figure S6) from the virome obtained during the Tara Oceans and Malaspina research expeditions (61,62), indicating that this dsDNA virus is a temperate phage. The MCP of Phm3 showed ∼30% sequence identity with the MCPs of characterized *Myoviridae* viruses.

### Capability to predict novel prophages

For the nine prophages predicted by Prophage Tracer, three of them may represent novel temperate phages (Table 2). The annotation of the potential capsid proteins of these prophages only showed remote homologs with other viruses (Supplementary Table S6). In particular, these three prophages were not detected as prophages (intact, incomplete or questionable) by PHASTER. As Prophage Hunter generated up to 106 ambiguous or inactive candidate prophage regions for the seven strains tested, we found that some ambiguous or inactive prophages partially overlapped with the three novel prophages predicted by Prophage Tracer. However, these hits either had low scores or were far away (> 10 kb) from the ones predicted by Prophage Tracer (Table 2 and Supplementary Table S5).

**Figure 3.**
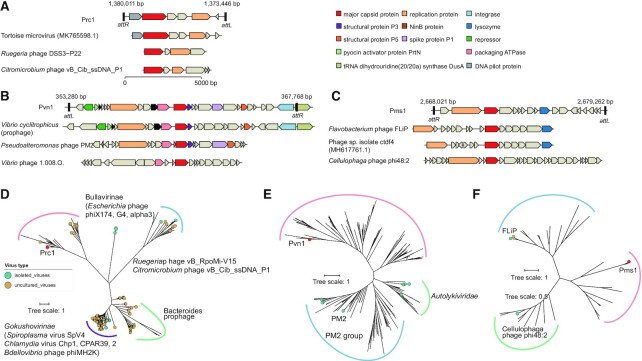
Gene maps and phylogenetic analysis of major capsid proteins of representative prophages. Gene maps of Pcr1 (**A**), Pvn1 (**B**) and Pms1 (**C**). Gene orientation of circular genomes was adjusted to make the aligned major capsid proteins. All the genomes are on the same scale as indicated. Genes are represented by block arrows and are colored according to gene function. Homologs of hypothetical proteins in (B) are indicated in black. Unrooted maximum likelihood trees of MCP homologs of Pcr1 (**D**), Pvn1 (**E**) and Pms1 (**F**). MCPs from isolated or uncultured viruses are highlighted in the trees, and MCPs from prophages are indicated as branches. Branch lengths are proportional to the number of amino acid substitutions.

Prophage Prc1 in *Ruegeria conchae* SCSIO 43209 has a 6 551-bp circular genome with nine predicted genes within the *Microviridae* family according to the genome content and phylogenetic analysis of MCPs (Figure 3AD). Closely related homologs of Prc1 MCP were found in other *Alphaproteobacteria* and metagenomic assembled *Microviridae* spp. and fell into a separate clade different from two members of the *Microviridae* subfamily (*Gokushovirinae* and *Bullavirinae*) and the recently identified *Ruegeriap* phage vB_RpoMi-Mini (63) and *Citromicrobium* phage vB_Cib_ssDNA_P1 (64). Temperate *Microviridae* phages are prevalent in the human gut and have been found to be integrated in the genomes of Firmicutes, Bacteroidetes, and Proteobacteria (65). Similarly, *Microviridae* sequences were dominant in coral virome communities (66), and their abundance increased in stressed/bleached corals (67,68). These results suggested that temperate *Microviridae* phages in coral are more diverse than previously thought.

In addition, prophage Pvn1 in *Vibrio nigripulchritudo* SCISO 43132 has a 14 465-bp circular genome with 27 predicted genes, integrated within the tRNA-dihydrouridine synthase A (*dusA*) gene and encoding double jelly roll (DJR) MCP. The genome organization of Pvn1 is similar to that of *Pseudoalteromonas* phage PM2 (69) and the recently identified prophages in *Vibrio* species (9) (Figure 3BE). Moreover, prophage Pms1 in *Mesoflavibacter sabulilitoris* SCSIO 43206 has an 11 220-bp circular genome with 18 predicted genes (Supplementary Table S6). BlastP searching revealed no sequence similarity of the phage structural genes to known viruses. Further utilization of the remote homology detection tool HHpred identified more phage-related genes in Pms1 (Figure 3C), especially INR78_12270, which showed undetectable amino acid sequence similarity but was structurally similar to the MCP of *Flavobacterium* phage FLiP (70), and INR78_12275, which showed 27% identity with the ssDNA replication protein in *Cellulophaga* phage phi48:2 (71). FLiP group phages are unusual lipid-containing ssDNA bacteriophages encoding DJR MCP that are mainly found in dsDNA bacteriophages (71,72). One representative FLiP group phage was isolated from red snapper tissue samples (73). All the MCPs found in the FLiP group primarily belong to marine Bacteroidetes, and the MCP of Pms1 was classified into a distinct clade different from other known FLiP group phages in the phylogenetic tree (Figure 3F). These results indicate that prophage Pms1 may represent a novel temperate bacteriophage that is similar to FLiP. Additionally, the Pmo1 element in *Marixanthomonas ophiurae* SCSIO 43207 is integrated into the tRNA gene and contains an integrase. This element contains various transporters, virulence associated protein E, VirE and outer membrane protein TolC encoding genes, and no phage structural genes were identified. This suggests that it may be other type of mobile genetic elements.

**Figure 4.**
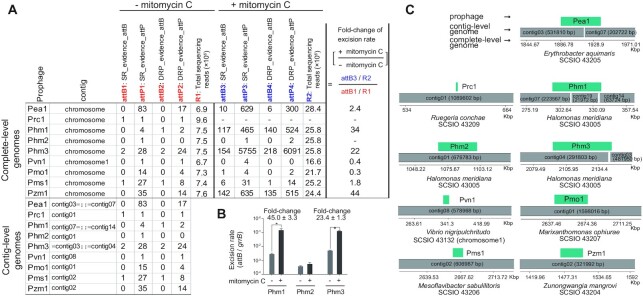
Prophage Tracer combined with qPCR to estimate the fold-change of prophage excision rate with or without mitomycin C. (**A**) Read counts in the outputs of Prophage Tracer of seven coral-associated bacterial strains with or without mitomycin C. SR, split read; DRP, discordant read pair. ‘–’ indicates ‘not detected’ or ‘unable to calculate’. The calculation of the fold-change of excision rate using read counts in the outputs of Prophage Tracer (if a zero is in the dividend, use one instead of zero). Prophage Tracer outputs using contig-level genomes are shown at left bottom. ‘::’ indicates a potential junction of two contigs. ‘ = contig’ indicates left junction and ‘contig = ’ indicates right junction. Full outputs including positions of *att* sites on each contig are shown in Supplementary Table S8. (**B**) Excision rates of Phm1, Phm2 and Phm3 prophages in SCSIO 43005 quantified by qPCR. Fold-change are indicated for Phm1 and Phm3, and significant changes are marked with one asterisk for *P* < 0.05. (**C**) Alignments of prophages to contig-level genomes.

Genomes of the seven coral-associated bacteria tested were complete-level genomes. To further evaluate the capability of Prophage Tracer to detect prophages using contig-level genomes, the contig-level genomes of the same seven strains were re-assembled using their corresponding short-read sequencing data. In contig-level genomes, an intact prophage may be integrated into an intact contig surrounded by host sequences, separated at the termini of two contigs, or assembled into their own contigs. For the above eight prophages and one mobile genetic element, we found that six were in one intact contig and three (Pea1, Phm1 and Phm3) were in the termini of two or three contigs (Figure 4A, C). For Pea1, Phm1 and Phm3, Prophage Tracer could detect almost identical split reads and discordant read pairs using either complete-level or contig-level genomes (Figure 4A and Supplementary Table S8). Furthermore, we tested Prophage Tracer using publicly available chromosome-level genomes that have their corresponding short-read sequencing data also deposited. A total of 81 candidate prophages or other mobile genetic elements with tyrosine-type recombinases or serine-type recombinases in 51 archaeal and bacterial genomes were predicted (Supplementary Table S2). Among them, 32 strains containing 48 prophage regions with high sequencing qualities were chosen for further assembling contig-level genomes. Using these contig-level genomes, Prophage Tracer predicted that 18 prophage regions were integrated into a contig and 15 were separated at the termini of two contigs. The remaining 15 prophage regions were not predicted in contig-level genomes, partly because they were assembled into their own separate contigs. These results indicate that our approach may be useful as a preliminary screening tool for prophages in contig-level genomes to determine whether it is worth converting contigs to complete-genomes in order to extract intact prophages for subsequent study.

Prophage Tracer can not only predict prophages using the above sequencing data derived from pure culture genomes, but also from data derived from enriched mixed culture. A recently discovered manganese oxidation bacterium ‘*Candidatus* Manganitrophus noduliformans’ cannot be isolated a pure culture, and can only be enriched in a mixed culture with other bacteria (74). Using the sequencing data of the mixed culture downloaded from NCBI, Prophage Tracer detected two potential prophage regions in ‘*Candidatus* Manganitrophus noduliformans’ with accurate boundaries (Supplementary Table S2). One potential region contains genes encoding typical phage structural proteins, suggesting that it is an active prophage. Another potential region does not contain phage genes but contain genes encoding conjugal elements, transposase, and defense systems (i.e. retron (75) and type 3 BREX system (76)), suggesting that it is a defense island. Taken together, our results indicated that Prophage Tracer, which is built-in database-independent, is a reliable tool for predicting novel prophages and other mobile genetic elements.

### Application and limits of Prophage Tracer to detect prophages

To further explore whether Prophage Tracer can be employed to detect prophage excision under stressed conditions, *H. meridiana* SCSIO 43005 was treated with 0.2 μg/mL mitomycin C for 4 hours and subjected to genome resequencing analysis. As shown in Figure 4A, compared to the untreated control, the number of extracted split reads and discordant read pairs containing the *att* sites of Phm1 and Phm3 relative to the total sequencing reads were much higher under mitomycin C induced condition. Our analysis on simulated data showed that the number of detected split reads of a prophage was highly correlated to the *att* site length at the same sequencing depth (Figure 2C), thus Prophage Tracer is not appropriate for the calculation of the excision rate of each prophage. However, for one specific prophage, read counts in the Prophage Tracer output can be used to estimate the fold-change of the excision rate under different conditions as shown in Figure 4A. It was found that mitomycin C induced the prophage excision of Phm1 and Phm3. Next, we performed qPCR to check the reliability of detecting the change of prophage excision using Prophage Tracer. Since Prophage Tracer can accurately predict the *att* sites of the prophages (Table 2), two pairs of qPCR primers were designed for each prophage to amplify the regions containing *attB* and *attP* (product size 200–300 bp; Supplementary Table S1), and used for quantifying the prophage excision. Consistently, qPCR results showed that the excision rates of Phm1 and Phm3 of SCSIO 43005 were greatly increased by mitomycin C (Figure 4B). The fold-change of excision rate quantified by qPCR was similar to the ones estimated using the reads values from the outputs of Prophage Tracer (Figure 4AB). The remaining six strains were also treated with mitomycin C and resequenced, and it was found that the excision rate of Pzm1 prophage of *Z. mangrovi* SCSIO 43204 was significantly increased with the mitomycin C treatment (Figure 4A). Thus, Prophage Tracer can be applied to detect the change of prophage excision at various conditions. Furthermore, the precise prediction of *att* sites by Prophage Tracer can then be used to design qPCR primers for subsequent quantification of the prophage excision rate by qPCR at a given condition.

Next, we investigated the detection power of Prophage Tracer to predict prophage with low excision rates and/or low replication rate at different sequencing depth. From our real sequencing data, Prophage Tracer can detect Phm1 (excision rate (*attB*/*gyrB*) of 2.6 × 10^−3^; replication (*attP*/*gyrB*) of 1.4 × 10^−2^), Phm2 (excision rate of 0.27 × 10^−3^; replication of 0.81 × 10^−3^) and Phm3 (excision rate of 4.2 × 10^−3^; replication of 1.3 × 10^−1^) using ∼290 × sequencing depth in the absence of mitomycin C (Figure 4). Prophage Tracer can also predict prophage that is not excisable but can replicate. As shown above, Pf4 prophage was not excised in the liquid culture of *P. aeruginosa* PAO1, but Prophage Tracer detected the presence of replicative form Pf4 based on the split reads containing *attP* at ∼170 × sequencing depth (Supplementary Table S4). Based on our analysis, in order to detect prophages with low excision rate, 100–1000× sequencing depth for a genome is recommended. At this range of sequencing depth, Prophage Tracer can detect the hidden prophages with excision rates (*attB*/*gyrB*) >10^−3^ and/or replication (*attP*/*gyrB*) >10^−3^ in host genomes. Otherwise, more efforts should be given to explore the special conditions that can trigger prophage activation or excision in order to detect the hidden prophages by Prophage Tracer.

Last but not the least, we wanted to explore whether Prophage Tracer missed any prophages with high excision rates in the seven coral-associated bacteria through the analysis of sequencing depth across genomes. Briefly, the presence of genomic regions with unusually high sequencing depth indicates the possible presence of a prophage in this region. As shown in Supplementary Figure S7, the regions containing the three prophages (Pea1, Phm3 and Pzm1) with high excision rate or replication showed unusually high sequencing depths were all predicted by Prophage Tracer. Indeed, one genomic region also showed high sequencing depth in strain SCSIO 43204 but it was missed by Prophage Tracer. Further analysis showed that this prophage encodes proteins similar with Gp1 (protease I), Gp29 (DUF935 family) and Gp36 (DUF1320 family) of Mu phages, suggesting that it is a Mu-like prophage capable of packaging host genomes with variable ends (Supplementary Table S6). Likewise, we used Prophage Tracer to reanalyze the phage DNA sequencing data of a published study in which three mitomycin C induced prophages, BLi_Pp2, BLi_Pp3 and BLi_Pp6 were experimentally identified in *Bacillus licheniformis* DSM13 (77). Prophage Tracer detected BLi_Pp3 and BLi_Pp6 but not BLi_Pp2 (Supplementary Table S2), and a previous study showed that prophage BLi_Pp2 can randomly package DNA of the host genome (77). Noticeably, sequencing depth of the six prophages in the seven coral-associated bacteria were indistinguishable compared with the rest of host genomes, but they were able to be captured by Prophage Tracer (Supplementary Figure S7).

Here, we showed that the power of detecting prophage by Prophage Tracer is limited by the nature of the prophage, either having a very low excision rate or having variable ends. Collectively, Prophage Tracer can detect hidden prophages if they can excise with stable *att* sites at excision rate higher than >10^−3^ at the sequencing depth of 100–1000 × with precise boundaries.

## DISCUSSION

Prophage-host interactions are currently recognized as being often mutualistic, rather than purely parasitic (78). Prophages are an important component of bacterial genomes and play critical roles in bacterial adaptation and evolution (7). The identification of active prophages is of central importance to the study of phage-host interactions. Prophage Tracer was validated and outperformed LUMPY using simulated reads, and it was determined to be superior to PHASTER and Prophage Hunter in predicting novel and highly divergent prophages in coral-associated bacteria. Furthermore, the predicted prophage boundaries were determined to be accurate, and read counts in the output can be used to estimate the fold-change of the excision rate under different conditions for one given prophage. The impact of prophage excision on genes containing *attB* or *attP* can also be manually analyzed in the Prophage Tracer output of overlapping split-read alignment. The accurate detection of prophage boundaries is important because prophages are usually integrated within bacterial functional genes (e.g. tRNA and tmRNA genes), and integration or excision may inactivate or reactivate target genes, which may affect the adaptation of bacterial hosts under diverse environments (7,79). Recent advances in DNA sequencing technologies have yielded overwhelming quantities of publicly available data on bacterial and archaeal genomes and their corresponding raw sequence reads. Mining active prophages in these genomes with accurate integrated sites may facilitate the study of phage ecology. Furthermore, we also expect that the application of Prophage Tracer will lead to the discovery of prophages in bacterial or archaeal taxa that are slow-growing and hard to cultivate, such as SAR11 (80) and ‘Asgard’ archaea (81). Additional functionality of the tool includes the identification of other families of mobile genetic elements that rely on site-specific recombinases, such as phage-inducible chromosomal islands, gene transfer agents, and integrative elements (82).

Because of the logic of Prophage Tracer, it has a few limitations. First, this tool cannot recognize prophages that do not excise or replicate during sample preparation for sequencing, or whose sequencing depth is too low to capture even one read containing *attB* or *attP*. Second, since Mu-like prophages excise with variable ends and other extrachromosomal/plasmidial prophages would not generate new junctions during their life cycle, they could not be detected by Prophage Tracer. Third, Prophage Tracer was designed for prophages with *att* site lengths shorter than read lengths. For *att* site lengths longer than the read length, discordant read pairs can also be used to estimate the boundaries. Lastly, Prophage Tracer may miss some prophages in contig-level genomes that have higher excision and replication activities and are assembled into their own separate contigs. In this case, the evaluation of sequencing the depth of contigs may be useful to distinguish which contigs are prophages. Therefore, Prophage Tracer is complementary to other tools, such as PHASTER and Prophage Hunter, and a combined approach would enable a more accurate prediction of prophages. Additionally, the performance of Prophage Tracer on long-read sequencing data has not been determined. Third-generation sequencing utilizing Pacific Biosciences (PacBio) and Oxford Nanopore Technologies (ONT) methods can generate long-read sequencing data, and these methods are now widely employed for genome sequencing. Further efforts to optimize the performance using long-read sequencing data could expand the application of Prophage Tracer.

In theory, circularized prophage sequences resulting from prophage genome excision can also be recognized by Prophage Tracer in metagenomic sequencing data. Several tools have been developed for the identification of viral sequences from assembled metagenomic data. Seeker recognizes bacteriophage genomes through deep learning utilizing Long Short-Term Memory (LSTM) models neural networks (83). DeepVirFinder also utilizes deep learning to identify viral sequences (84). VirFinder employs *k*-mer frequency and machine learning to distinguish viral from bacterial contigs (85). Excised prophages could be an important component of the virome in various ecosystems (4,10). These tools cannot detect viral contigs representing excised circular or linear prophage DNA unless this prophage is excised or replicates at a high enough rate to assemble a viral contig. Prophage Tracer may recognize rare prophage excision signals in the metagenome if the host genome can be assembled. In this case, Prophage Tracer could be complementary to other current state-of-the-art tools for the study of prophages in metagenomes.

## DATA AVAILABILITY

The code for the Prophage Tracer is written in the shell script including the Unix awk utility and is publicly available (https://github.com/WangLab-SCSIO/Prophage_Tracer). Bacterial genomes and sequencing read data have been deposited under GenBank BioProject numbers PRJNA668462 and PRJNA682846.

## Supplementary Material

gkab824_Supplemental_Files
