# Supplementary material for: Prophage Tracer: precisely tracing prophages in prokaryotic genomes using overlapping split-read alignment
Source: Nucleic Acids Res. 2021 Sep 22;49(22):e128. doi: 10.1093/nar/gkab824 (PMC8682789; doi:10.1093/nar/gkab824)
Supplement: gkab824_Supplemental_Files [file gkab824_supplemental_files.zip › 20210816 Supplementary figures-Prophage Tracer.pdf]

Supplementary materials

Prophage Tracer: Precisely tracing prophages in prokaryotic genomes using overlapping split-read alignment

Kaihao Tang[1,2], Weiquan Wang[1,2,3], Yamin Sun[4], Yiqing Zhou[1,2,3], Pengxia Wang[1,2,3], Yunxue Guo[1,2,3], Xiaoxue Wang[1,2,3*]

1. Key Laboratory of Tropical Marine Bio-resources and Ecology, Guangdong Key Laboratory of Marine Materia Medica, Innovation Academy of South China Sea Ecology and Environmental Engineering, South China Sea Institute of Oceanology, Chinese Academy of Sciences, No.1119, Haibin Road, Nansha District, Guangzhou 511458, China

2. Southern Marine Science and Engineering Guangdong Laboratory (Guangzhou)，No.1119, Haibin Road, Nansha District, Guangzhou 511458, China

3. University of Chinese Academy of Sciences, Beijing, China

4. Research Center for Functional Genomics and Biochip, 23 Hongda St., Tianjin 300457, China

[*]Correspondence should be addressed to: xxwang@scsio.ac.cn

Keywords: prophage, prediction, split read, DNA sequencing raw data

Running head: Prophage Tracer for prophage prediction

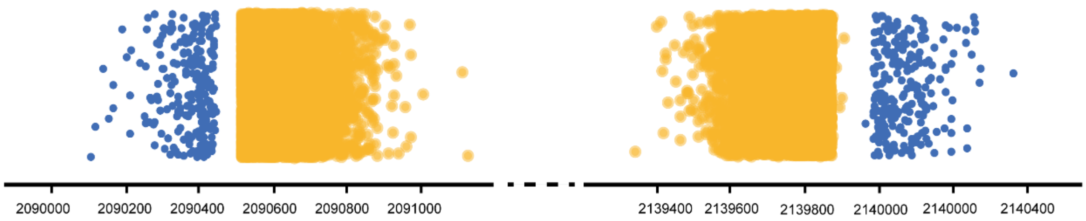

Figure S1. Scatter plot using POS and MPOS strings of extracted discordant read pairs to support the active Phm3 prophage (2090511-2140009) of *Halomonas meridiana* SCSIO43005 when induced by mitomycin. Blue and yellow dots indicate discordant read pairs supporting *attB* and *attP* events, respectively.

A

```
Query_126135  1        TGCCGCTTTTCCGTTGCACCCATGCTCGATGGGACTCACTATTAAGGTTCTATAAGGCTT  60
0             353280               .....G..........................................  353327
0             367733   .............................CT.....                          367768

Query_126135  61       GATAGTGAGTCATACGTACTATATCCGTATTTCGTTACTACGAAGCCACACAAGTTGCCA  120
0             353328   ............................................................  353387

Query_126135  121      TTACGGCACTATTATCTGTATTGACTAGTT  150
0             353388   ..............................  353417
```

B

```
Query_11  1        GTCTATGGATAGTAATATGGCGTAAGTTATTGATTTTACGTTGTTCAAGGTAAGAAAAAA  60
0         1501844  ............................................................  1501903
0         1538077                        .....C..........A.C.A.......C..T......  1538114

Query_11  61       GACGTCCTGAGACGTCTTCGTTCTTATATTTGGTGGGGCGGCGGGACTTGAACCCGCGTC  120
0         1501904  ...........................................                   1501946
0         1538115  ........AG..............-...................................  1538175
                                              \        \
                                              |        |
                                              C        A

Query_11  121      CAGAAAACCTACATCCAAGGCGCTACATGCT  151
0         1538176  ...............................  1538206
```

Figure S2. Examples of overlapping split-read alignment generated by BlastN. (**A**) The alignment of a split read containing *attP* of Pvn1 in *Vibrio nigripulchritudo* SCSIO 43132 shows the mismatch. (**B**) The alignment of a split read containing *attB* of CP4So in *Shewanella oneidensis* MR-1 shows the deletion of a ‘A’, which resulted in deletion of a U at the 3′-end of SsrA, destroying this wobble G·U base pairing.

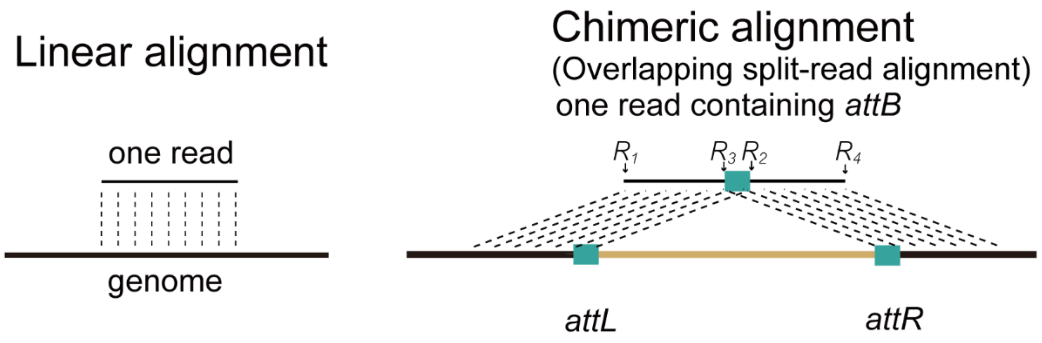

Figure S3. Schematic diagrams of a linear alignment and a chimeric alignment. R_1-R_2 and R_3-R_4 are positions of two linear alignment in a chimeric alignment on the query read.

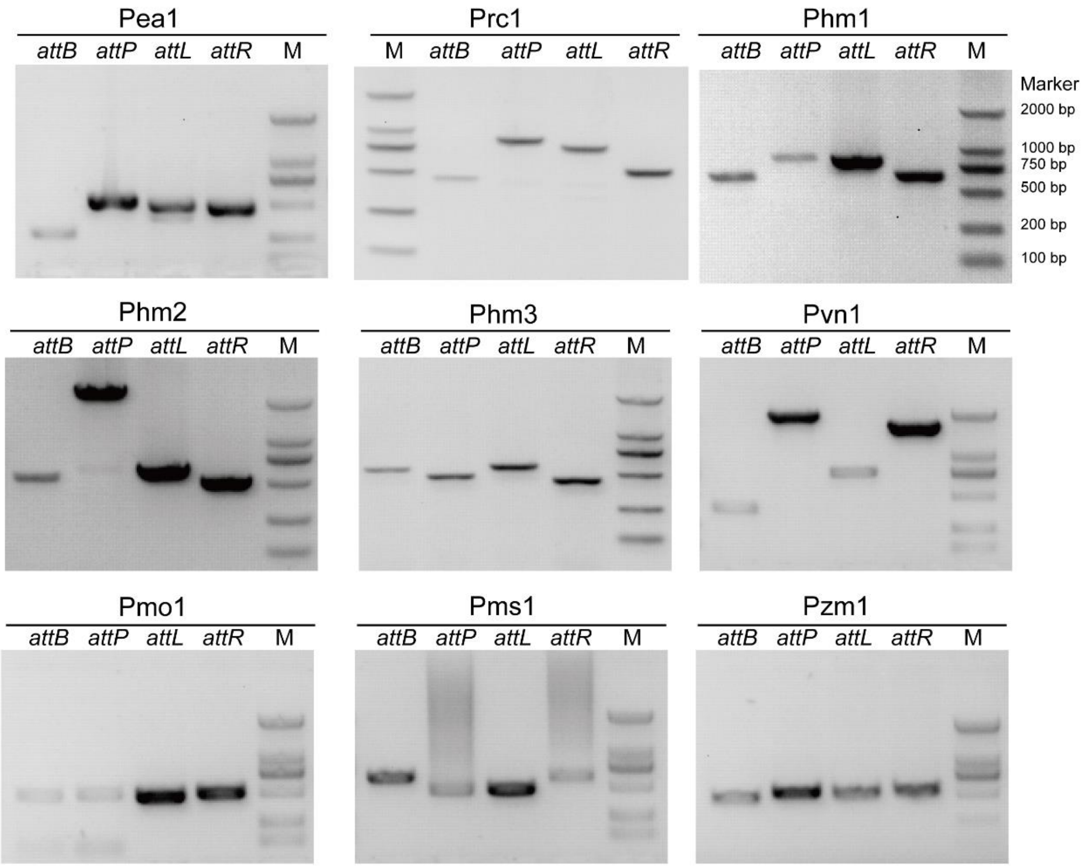

Figure S4. Verification of predicted active prophages of coral-associated bacteria by a PCR-based assay followed by sequencing. The additional product (~3000 bp) in Phm2-attP was a nonspecific amplification confirmed by sequencing.

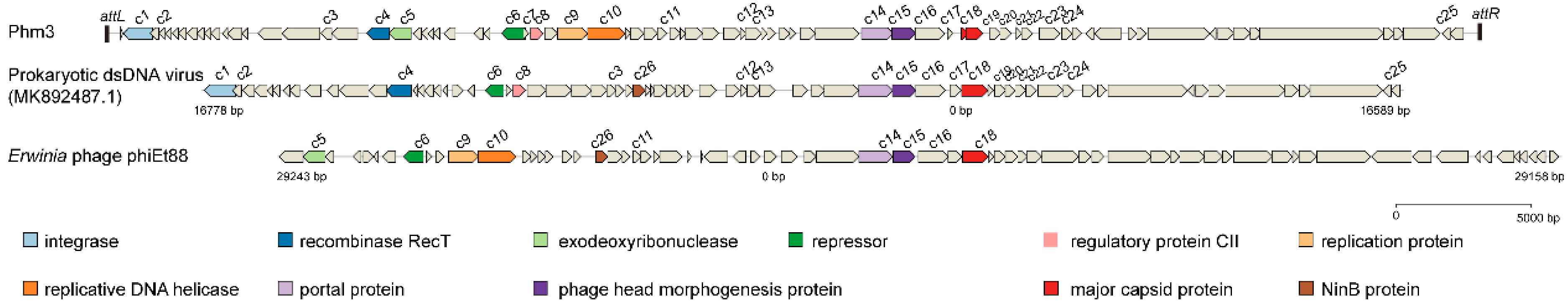

Figure S5. Gene maps prophage Phm3 and other related phages. They are generally syntenic and share some structural genes, including major capsid protein (MCP). The ORF of MCP in Phm3 is truncated.

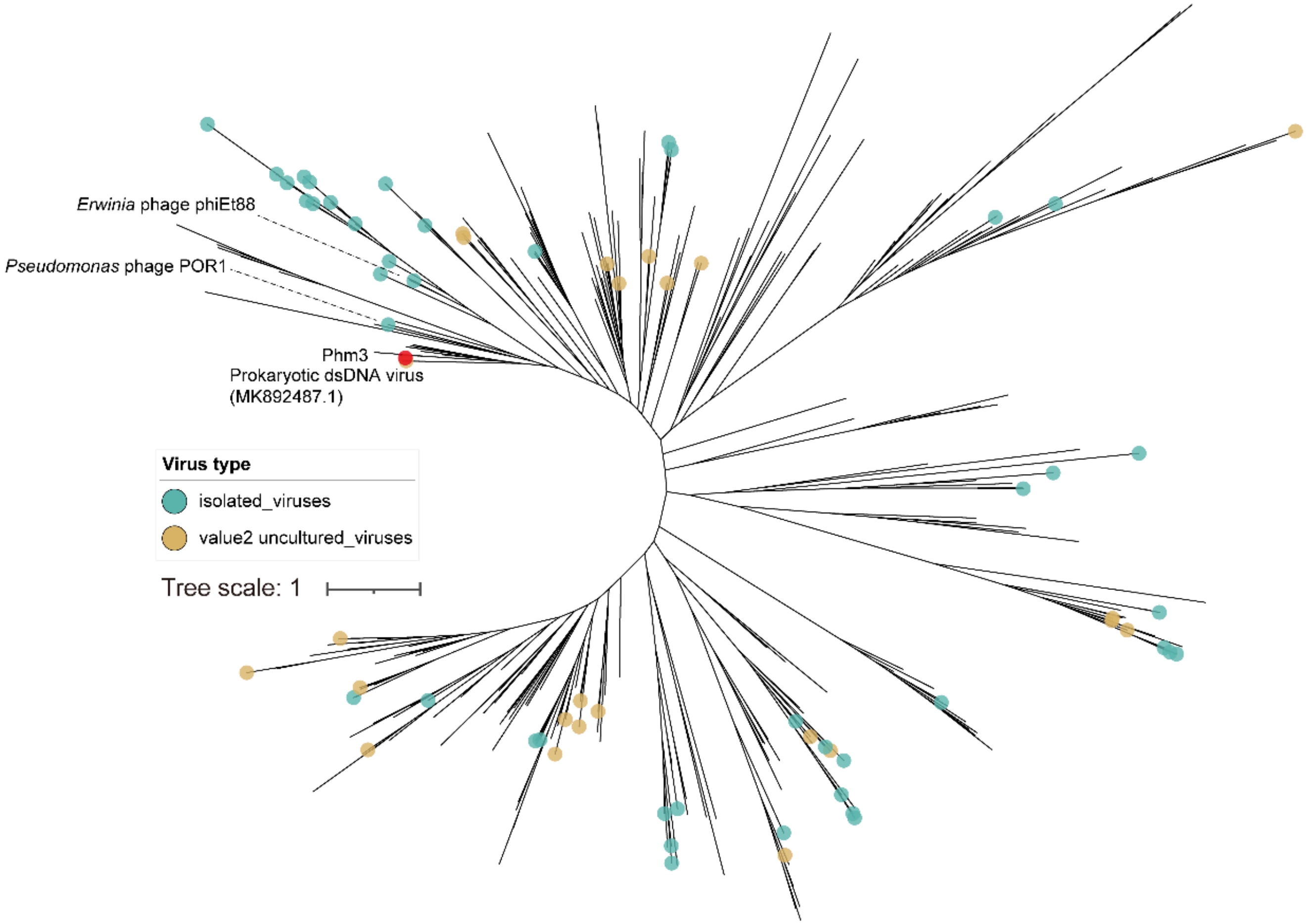

Figure S6. Unrooted maximum likelihood tree of MCP homologues of prophage Phm3 and other related phages. MCPs from isolated or uncultured viruses are highlighted in the trees and MCPs from prophages are indicated as simple branches. Branch lengths are proportional to the number of amino acid substitutions.

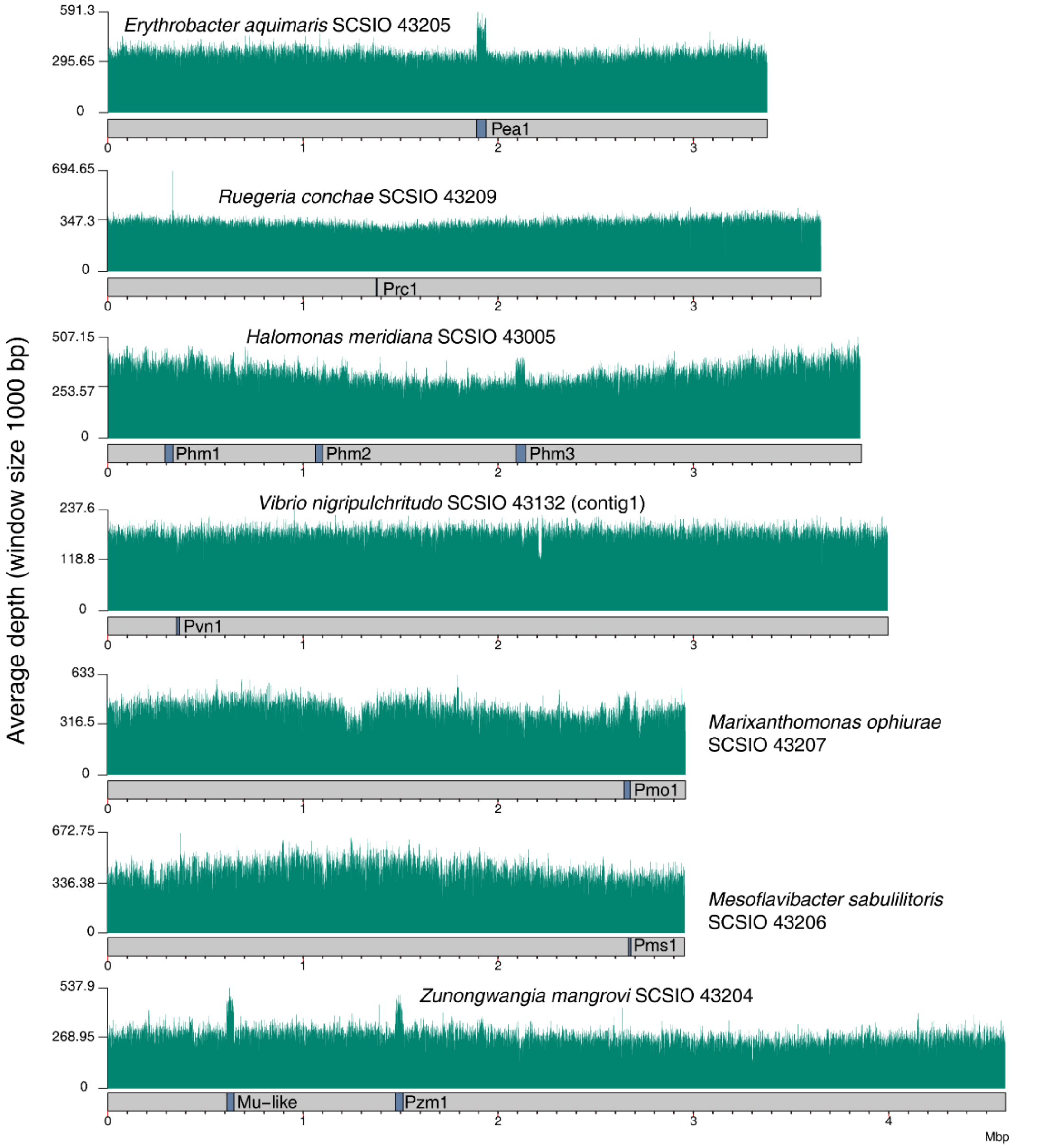

Figure S7. Sequencing depth across genomes of seven coral-associated bacteria. Average depth was calculated by an R/Bioconductor package karyoploteR. Note: since no prophage or unusually high depth peak is found in the second chromosome (contig 2) of *Vibrio nigripulchritudo* SCSIO 43132, only contig 1 was shown here.
