# Supplementary material for: Prophage Tracer: precisely tracing prophages in prokaryotic genomes using overlapping split-read alignment
Source: Nucleic Acids Res. 2021 Sep 22;49(22):e128. doi: 10.1093/nar/gkab824 (PMC8682789; doi:10.1093/nar/gkab824)
Supplement: gkab824_Supplemental_Files [file gkab824_supplemental_files.zip › Supplementary_Table_legends.docx]

**Supplementary Table legends**

**Table S1. Primer pairs used for confirming prophage induction**

**Table S2. Predicted prophages or other mobile genetic elements from publicly available genomes and sequencing data.** Prophages both detected by Prophage Tracer and PHASTER are highlighted in blue. "N/A" indicates no prophages were predicted.

**Table S3. Evaluation the performance of Prophage Tracer and LUMPY on predicting prophages with diverse att site length in simulated genomes.** Because of using random bases, a part of predicted *att* site positions shift slightly with simulated sites. Expected split reads in the SAM file using simulated data was extracted simply accroding to CIGAR strings of *a*M*b*S or *a*S*b*M (integer values of a and b from 1-149) mapping at expected prophage positions. Please refer to VCF specification for LUMPY outputs in the right side cells.

**Table S4. Prediction of known active prophages in representative strains by Prophage Tracer**

**Table S5. Full table of Table 2.** Full outputs of Prophage Tracer, LUMPY, PHASTER and Prophage Hunter.

**Table S6. Annotation of predicted active prophages in coral-associated bacteria by Prophage Tracer**

**Table S7. Att sites of predicted active prophages in coral-associated bacteria by Prophage Tracer**

**Table S8. Full outputs of Prophage Tracer, LUMPY, PHASTER and Prophage Hunter using contig-level genomes**
